# Outcome Measures in Cancer Rehabilitation: Pain, Function, and Symptom Assessment

**DOI:** 10.3389/fpain.2021.692237

**Published:** 2021-09-29

**Authors:** Eduardo Maldonado, Nirguna Thalla, Sargoon Nepaul, Eric Wisotzky

**Affiliations:** ^1^MedStar National Rehabilitation Hospital, Washington, DC, United States; ^2^Department of Rehabilitation Medicine, Georgetown University, Washington, DC, United States

**Keywords:** outcome measures, cancer rehabilitation, pain, function, rehabilitation

## Abstract

Assessment of cancer rehabilitation outcome measures is integral for patient assessment, symptom screening, and advancing scientific research. In the broad field of cancer rehabilitation, outcome measures can cross-cut across many different branches of oncologic care including clinician-reported, patient-reported, and objective measures. Specific outcome measures that apply to cancer rehabilitation include those pertinent to pain, function, quality of life, fatigue, and cognition. These outcome measures, when used in cancer rehabilitation, can be utilized to evaluate the effectiveness of an intervention and to triage to the appropriate supportive care service. This review article summarizes some of the commonly used outcome measures that can be applied in the cancer rehabilitation setting to support scholarly work and patient care.

## Introduction

Living life post cancer diagnosis is becoming a reality in the United States and across the world for a growing number of patients. This is in large part due to the advancements in cancer disease, specific knowledge, screenings, and treatments. It is expected that by the year 2040, there will be more than 26 million cancer survivors in the United States (1). This growing population will result in an increase in the demand for specialists who will be tasked to address the increasing burden of the devastating complications associated with cancer. These not only include a variety of functional physical impairments but also extend to emotional, social, psychological, and cognitive stressors that can impact the overall quality of life of a patient. The current rate of cancer-related disabilities remains exceedingly high with the demands for even readily treatable physical conditions being met at a rate of 1–2% (2).

For these patients, alleviating the impact of physical, social, psychological, cognitive, and emotional burdens of the disease is paramount to improving their quality of life and function. Enabling the patient to achieve this is the goal of cancer rehabilitation. The field of cancer rehabilitation can be divided into four separate categories based on the temporal course of the disease (3), which includes preventative, restorative, supportive, and palliative rehabilitation. Preventative rehabilitation seeks to control the outcomes prior to diagnosis or cancer-related interventions to maximize functionality early in the treatment course. Restorative rehabilitation aims to maximize recovery in those undergoing treatments and having existing impairments. Supportive and palliative rehabilitation tend to focus on disease progression and declining function (3). These therapies are geared toward augmenting self-care ability and mobility and relieving distressing symptoms, such as pain, fatigue, and anorexia. Cancer rehabilitation can also be tailored to address system-specific, disease-specific, and symptom-specific problems. Specialists need to track the outcomes of the interventions used to address these problems. Data achieved through outcome measures is a primary vehicle in medicine to assess the quality of interventions. In a growing field such as cancer rehabilitation, a prudent understanding of these measures will create a foundation from which to develop.

In this review, we explore a variety of outcome measures used in cancer rehabilitation and the related fields. In the modern world of medicine and evidence-based treatments, every specialty needs to have focused assessments of the measures they use to analyze treatment effectiveness. Without a proper understanding of the appropriate outcome measures, it is impossible to gauge the effectiveness of the outcomes of treatments that are being validated by these measures. This is the critical first step and is consistent with a growing national trend on the use of defined values. Current research on outcome measures specific to cancer rehabilitation is limited. Creating a better understanding of the validity, scope, and action ability of these measures will allow providers to get a better sense of when to utilize specific treatments, an understanding of how effective they may be, and how they can fit into the overall patient-care goals. Creating this foundation increases the confidence of the providers and emphasizes the need for quality-based, evidence-based care. In this review article, we organize and assess the utility of specific outcome measures, commonly seen under the broader umbrella of cancer rehabilitation, such as function, quality of life, pain, fatigue, cognition, and objective measures. Please note that this review does not encompass all the pertinent and available outcome measures that can be used in cancer rehabilitation, but presents a starting point for commonly used measures.

An outcome measure is a tool, usually in the form of a questionnaire, used to reflect the impact of a healthcare service or intervention on the health status of a patient (29). Outcome measures may be used to determine the baseline function of a patient. Similarly, the same instrument can be used to determine the progress and efficacy after a certain intervention (30). Therefore, outcome measures are often used to assess the response to treatment.

## Methods

This review discusses some of the more commonly used outcome measures in the field of cancer rehabilitation, specifically, those that pertain to general function, fatigue, pain, quality of life, cognition, and objective measures. We provide the following six key elements that help describe the properties of each measure:

- **General description:** includes the definition and purpose of the measure
- **Psychometric properties:** include a combination of validity, reliability, internal consistency, test-retest reliability, and ceiling/floor effects
- **Burden:** indicates the number of items in and the time taken to complete the questionnaire
- **Scoring:** outlines how the measure is scored
- **Scope:** includes any domain or subdomain that may be a part of the outcome measure (for example: if mobility is being assessed – are transfers, ambulation, and stairs part of this measure?)
- **Clinical relevance:** outlines how the measure can be most useful in a clinical setting.

The outcome measures selected in each section were chosen based on a careful review of the cancer rehabilitation literature, discussion amongst the authors of this paper, and discussion with cancer rehabilitation experts from other institutions.

### Pain Outcome Measures

The frequency, severity, and impact which pain has on the quality of life of patients living with cancer are important factors to be considered by the clinician (17). Formal instruments have been developed to help describe and measure pain, thereby helping clinicians and patients track the progression of pain or response to treatment. We focus on five commonly used outcome measures in Table 1, which include the Brief Pain Inventory (BPI), the Shoulder Pain and Disability Index (SPADI), Quick-Disability of the Arm, Shoulder, and Hand (Quick-DASH), the Pain Disability Index (PDI), and the McGill Pain Questionnaire (MPQ).

**Table 1:** Comparison of five common cancer pain outcome measures.

	**BPI**	**SPADI**	**Quick-DASH**	**PDI**	**McGill Pain questionnaire**	**TNS**
General Description of measure	It is a patient-reported outcome measure that assesses pain intensity and interference with various areas of function (4).	It is a self-administered questionnaire that assesses pain and disability related to shoulder problems (5–7).	It is a self-reported questionnaire that looks at the ability of a patient to perform certain upper extremity activities (8).	It is a short self-report that measures the impact that pain has on the ability of a person to participate in essential life activities (9).	It is a self-reported tool for measuring the multidimensional aspects of the pain experience (10–13). A long form and a short form exist; a revised version of the SF-MPQ was created to assess both neuropathic and non-neuropathic pain (14, 15).	The TNS is used to assess and quantify chemotherapy-induced peripheral neuropathy (CIPN) (16).
Reported psychometric properties	Reliable and valid for research purposes (17). High internal consistency, and excellent test-retest reliability of the two domains of the BPI (18, 19).	High internal consistency with Cronbach α, as well as good construct validity, correlating well with other region-specific shoulder questionnaires (5, 7). No large floor or ceiling effects have been observed (5, 7).	Internal consistency of the questionnaire has shown Cronbach alpha scores in the good and excellent ranges of 0.87 and 0.92 in two separate studies (20, 21).	Reliable and valid self-report indicator of general pain-related disability (22, 23). Has modest test-retest reliability, is internally consistent, and is able to discriminate between patients with low and high levels of disability (9, 23, 24).	Both forms have been shown to be psychometrically sound, valid, and reliable instruments with good discriminative capacity (10, 12, 15). Furthermore, the SF-MPQ-2 total and scale scores have demonstrated good-to-excellent internal consistency, as well as reliability and validity in a veteran population with chronic pain (14).	The TNS clinical versions have been tested in a number of settings where construct validity has been demonstrated (25). These versions also likely represent a more sensitive measure of CIPN condition than the FACT/GOG-Ntx. The mTNS correlates with balance, physical performance, and quality of life, and discriminates between cancer and healthy controls (16).
Most common burden for clinical practice	No training is required to answer the form, which takes about 5 min to complete (18, 26).	No training is required to answer the form, which takes about 5–10 min to complete (5, 6).	It has been designed to be efficient and easy for completion with a patient population of any educational level.	The PDI consists of 7 questions. The mean administration time varies between 1 and 2 min (24).	The LF-MPQ takes about 20 min to complete and contains complex vocabulary, which some patients find difficult to understand (10). The SF-MPQ takes about 2–5 min to complete and has simpler vocabulary.	There are various versions of the TNS. All of them are limited by their inability to properly assess neuropathy-related pain severity and the burdensome nature of the test.
General scoring guidelines	Short form consisting of two domains: 4 pain severity items and 7 pain interference items, each rated on a 0–10 scale (4, 18). There is also a question regarding percent of pain relief by analgesics and another one about pain localization.	13-item self-report questionnaire (5). The first dimension consists of 5 questions regarding the individual's pain severity. The second dimension consists of 8 questions regarding the individual's degree of difficulty with various activities of daily living that require upper extremity use. The mean of the two subscales is averaged to produce a total score from 0 (best) to 100 (worst).	The tool utilizes a 5 point likert scale. Higher scores indicate a greater level of disability. The score ranges from 0 (no disability) to 100 (most severe disability).	7-item questionnaire that uses a 10-point scale ranging from 0 (no disability) to 10 (total disability) to rate the degree to which pain interferes with those 7 items (23). A total score ranges from 0 to 70. The higher the score, the greater the disability related to pain (9).	The SF-MPQ contains 15 word descriptors that describe the sensory and affective dimensions of pain and are rated on an intensity scale as 0 = none, 1 = mild, 2 = moderate or 3 = severe (10, 12). Scores range from 0 (no pain) to 78 (severe pain) (13).	The tool evaluates neuropathy signs and symptoms and incorporates nerve conduction study results (16). It assesses the presence, characteristics, and location (distally versus proximally) of symptoms, as well as the presence, severity, and location of several physical findings (16). A physician or nurse scores each neuropathy item on a scale of 0–4. The scores are summed to obtain a total score (16).
Clinical relevance	Used in patients suffering from chronic pain, cancer-related pain, osteoarthritis, fibromyalgia, depressive disorders, and in research (18, 19)	Used in patients suffering from musculoskeletal conditions, joint pain and fractures, chronic pain, among others (6, 7). A higher score in the SPADI questionnaire indicates greater impairment or disability (7).	Quick-DASH has proven to be versatile with excellent scope in the setting of upper extremity musculoskeletal conditions and chronic pain and has been applied to workman's compensation, sports and musician related injuries (27).	Can be used to evaluate patients initially, to monitor them over time, and to judge the effectiveness of interventions (9). Moreover, it can be used for all diagnoses in which pain is a disabling factor (24).	The total score of the SF-MPQ correlates highly with the LF-MPQ in patients with chronic pain due to cancer (10). Similarly, the LF-MPQ is a valid measurement of pain in the cancer population (10).	The TNSc may be a reliable method for assessing the severity as well as changes in CIPN (28). Furthermore, some evidence exists that the TNS is responsive to changes over time in patients receiving higher and higher cumulative neurotoxic chemotherapy doses (16).

*BPI is the Brief Pain Inventory; SPADI is the Shoulder Pain and Disability Index; DASH is the Disability of the Arm, Shoulder, and Hand; PDI is the Pain Disability Index; SF-MPQ is the Short-Form-McGill Pain Questionnaire; and TNS is the Total Neuropathy Score*.

### General Functional Outcome Measures

Monitoring patient function prior to, during, and after cancer treatment is an essential function of cancer rehabilitation. Tracking function over time is an important way to assess how patients are progressing with rehabilitation. Functional outcome measures, such as the Functional Assessment of Cancer Therapy-General (FACT-G), Eastern Cooperative Oncology Group (ECOG), Karnofsky Performance Scale (KPS), and Common Terminology Criteria for Adverse Events (CTCAE) are several widely utilized outcome measures of general function that provide objective data that clinicians utilize before making treatment decisions and assessing the response to cancer and rehabilitation treatments. In Table 2, we break down each of these measures to better understand their utility and quality.

**Table 2:** Comparison of five common cancer functional outcome measures.

	**FACT-G**	**ECOG**	**KPS**	**CTCAE**	**MDASI**	**Promis PF Cancer SF**
General Description of measure	27 questions to assess four domains in cancer patients: physical well-being, social-family well-being, emotional well-being, and functional well-being (32).	Performance status measure used to plan treatment trials. Used to track changes in a patient's level of functioning and compare the effectiveness of oncologic therapies (33).	Patient's functional status as an 11-point scale correlating to percentage values ranging from 100% (no evidence of disease, no symptoms) to 0% (death) (34)/	Set of criteria that are used for adverse event reporting of cancer therapy (35).	Assesses the severity of the most common symptoms and interference of these symptoms with daily living for cancer patients (36, 37).	Patients reported outcome measures for several domains including Physical Function. Cancer expert reviewers utilized a larger pool of PROMIS data to develop a form of clinically relevant items to assess physical function in cancer populations (38).
Reported psychometric properties	Cronbach Alpha: 0.89–0.9 (39)	Kappa is used to evaluate non-chance agreement. Kappa was 0.44 (0.38–0.51) (40, 41).	Cronbach Alpha: 0.97 (42)	Patient reported outcome component studied (PRO-CTCAE). ICC: 0.76 (43)	Cronbach Alpha: 0.89–0.92 (25)	Cronbach Alpha: 0.92–0.96 (38).
Most common burden for clinical practice	Completing is simple and intuitive	No subscales and no scoring algorithm, this scale has very low burden	Simple and rapid for the health care provider	Utilizers needs to consider a library of items representing 790 discrete adverse events	Easy to understand, takes 2–5 min to complete (36, 37).	Can be completed online or using paper assessments. Short forms are patient friendly and typically take 5–10 min to complete
General scoring guidelines	Symptom assessment is graded on a 5 point scale from 0 (not at all) to 4 (very much) (44).	Scored on a six-point scale of performance status (PS) 0–5. It ranges from 0 (fully active) to 5 (dead) (45).	Three states (conditions). A: normal activity and work B: abnormal activity, can self-care, C: inability to perform self-care.	In general, Adverse event severity is graded from 1 to 5. Mild (Grade 1) to Death (Grade 5).	11-point scale, 0 = no symptom and 10 = highest symptom severity (23). Interference scale is 0–10, 0=did not interfere; 10=completely interfering (23).	Scoring is done using a T-score metric. Defined by how it compares to the scores of the reference population. Higher score equals more of the domain measured (46).
Clinical relevance	Assessment tool that can be utilized in a variety of clinical settings, especially those undergoing active therapies. Disease-specific forms are available.	Determination of whether patients receive or don't receive oncologic treatments. Has been shown to correlate with survival in many cancer forms (47)	Assesses the need for a certain amount of custodial care, or dependence on medical care in order to continue to live (34).	Provide standardization for the description and exchange of safety information in oncology research (19, 20).	Categorization of symptom variety and severity and understanding of a patient's daily living functions (25). Disease-specific forms are available.	Intended to outperform classic tools for patient outcomes. Utilizes Item Response Theory models. Can use item response to predict scores, expected answers to different items and to improve overall precision (46).

*FACT-G is the Functional Assessment of Cancer Therapy-General; ECOG is the Eastern Cooperative Oncology Group; KPS is the Karnofsky Performance Status; CTCAE is the Common Terminology Criteria for Adverse Events; MDASI is the MD Anderson Symptom Inventory; Promis PF Cancer SF is the Promis Physical Function Cancer Short-Form*.

### General Quality of Life Measures

The assessment of the quality of life (QOL) has become one of the most critical parts of oncologic care. It is common that decisions to initiate, avoid, and cease treatment may be based on a discussion regarding QOL of the patient. In addition, QOL has become an important measure of the success (and failure) of the aspects of oncologic treatment. Therefore, familiarity with various QOL measurement tools is essential in oncology care. While different QOL measures exist, in Table 3, we review the Short Form-36 (SF-36), European Organization for Research and Treatment of Cancer (EORTC), and National Comprehensive Cancer Network -Distress Thermometer (NCCN-DT).

**Table 3:** Comparison of three common cancer quality of life measures.

	**MOS SF-36**	**EORTC-QLC**	**NCCN-DT**
General Description of measure	36 Item short Form Health Survey Questionnaire. Contains two components: Mental health and physical health (48, 49).	Cancer specific questionnaire, for physical and psychological symptoms (50).	Screening tool to identify potential sources of distress (51).
Reported psychometric properties	Cronbach alpha: 0.7 (49, 52).	Cronbach alpha: 0.62–0.90 (53).	Cronbach alpha: 0.82–0.90 (51).
Most common burden for clinical practice	10 min to complete (48).	Nine multi-item scales, which takes more time to complete than many other quality of life measures (54).	Inadequate psychosocial staffing once a patient comes in with a low score (<3) (51).
General scoring guidelines	Numerical scores range from 0 to 100. Mean of 50. Standard deviation of 10 (49).	Numerical scores range from 0 to 100 for each section of the measure. Higher score represents a better level of functioning (55).	Single item tool using 0 (no distress) to 10 (extreme distress) (56)
Clinical relevance	Widely used. Translated and adapted for use in more than 50 countries (52).	Beneficial for routine care, as they cover both symptoms and the impact on functioning (50, 54).	Brief tool with problem list that requires 2.5 min to complete. Can be used in every visit (57).

*MOS SF-36 is the medical outcome study 36-item short form survey; EORTC-QLC is the European Organization for Research and Treatment of Cancer-Quality of Life Questionnaire; and NCCN-DT is the National Comprehensive Cancer Network-Distress Thermometer*.

### Fatigue Outcome Measures

Cancer-related fatigue is a common experience among cancer survivors. It is estimated that the predominance of this symptom is close to 48% and may increase with disease burdens, such as metastasis, or treatment, such as chemotherapy (31). A significant variable driving the assessment and treatment of cancer-related fatigue has been the recognition of its negative effect on the quality of life (31). Various scales have been used to objectively measure fatigue in both the research and clinical settings. In Table 4, we present three outcome measures: the Patient-Reported Outcomes Measurement Information System (PROMIS) Fatigue Short Form, the Modified Brief Fatigue Inventory (MBFI), and the Visual Analog Scale to Evaluate Fatigue Severity (VAS-F).

**Table 4:** Comparison of three common cancer fatigue outcome measures.

	**PROMIS Fatigue short form**	**Modified brief fatigue inventory (MBFI)**	**Visual analog scale to evaluate fatigue severity (VAS-F)**
General Description of measure	Set of person-centered measures that evaluates and monitors physical, mental, and social health in adults and children (58, 59)	It is a questionnaire used to measure the intensity and frequency of fatigue in cancer patients (60).	The VAS-F is a scale used to measure the severity of fatigue. It was designed to be a simple and quick measure of fatigue and energy levels for patients in the general medical population (61).
Reported psychometric properties	Good internal consistency, reliability, as well as evidence for convergent and concurrent validity (62).	Excellent test-retest reliability, Spearman rank coefficient (*r*) of 0.800 (*P* < 0.001) (60). Cronbach's α of 0.938. Discriminant validity and adjusted discriminant validity were also found to be significant.	High internal reliability ranging from 0.94 to 0.9635 (63). Some have criticized the scale as ambiguous, suggesting that it is not sensitive to the distinction between fatigue and sleepiness (61, 64, 65)
Most common burden for clinical practice	This form can be administered through an iterative computer adaptive testing (CAT) system or via paper form (59). May take 5–15 min to administer, depending on the specific form used (59).	9-item survey measuring the core facets of functioning and quality of life related to fatigue. Comprehensive, yet simple design.	The scale consists of 18 questions on a 10 cm line relating to the subjective experience of fatigue. It should take <5–10 min to complete. The VAS-F is simple to administer and requires little time for completion (66).
General scoring guidelines	Items are scored numerically for an individual's response to each question. Scores are added and the total raw score is converted to a T-score (59). Higher scores mean more of what is being measured, for example more fatigue (59).	Each item in the MBFI contains a numeric rating scale. Questions assess fatigue over a 7-day period. Items are on a 1–7 scale, with 1 representing “none of the time” and 7 representing “all of the time.” The overall score is simply the arithmetic mean of the 9 items (60).	Respondents choose a number between 1 and 10 for each item, representing how they currently feel, along a visual analog line that extends between two extremes (e.g., from “not at all tired” to “extremely tired”). A fatigue severity score is calculated as the mean of the 13 items in the fatigue subscale, with higher scores indicating higher levels of perceived fatigue (64). The remaining 5 items are averaged to produce an energy subscale score that ranges from 0 to 10, with higher scores indicating higher levels of energy (63).
Clinical relevance	PROMIS Cancer Fatigue Short Form is a reliable and valid measure of fatigue in cancer patients (58).	Comorbidity and cancer stage have been shown to be significant predictive correlates of MBFI scores (60). The MBFI is a comprehensive yet simple design, which makes it ideal for the clinical setting in the context of initial assessment in addition to post treatment surveillance (60).	The VAS-F is a simple instrument that may be used when measuring fatigue and energy as the outcome variables of interest (66). Potential uses include assessments of fatigue before and after clinical interventions as an indication of the effectiveness of therapy (66). However, the ability to act as an outcome measure sensitive to change with disease progression or treatment is unknown (67).

*PROMIS is the Patient-Reported Outcomes Measurement Information System; MBFI is the Modified Brief Fatigue Inventory; and VAS-F is the Visual Analog Scale to Evaluate Fatigue Severity*.

### Cognitive Outcome Measures

Impaired cognition is a common issue reported in patients undergoing cancer treatment as well as beyond treatment. Many factors have been proposed to impact cognition in cancer, including various cancer treatments, mood disorders, fatigue, and poor sleep. Given how pervasive these symptoms can be, it is important to assess and monitor cognitive function during and after cancer treatment. In Table 5, we review the Montreal cognitive assessment (MoCA) and the FACT-cognitive function (FACT-COG). While FACT-COG is designed specifically for cancer survivors, it should be noted that there is no gold standard cognitive assessment for the cancer population. Overall, it is important to consider that all cognitive screening measures carry a risk of false-positive errors, particularly when used with individuals whose education level and/or cultural and linguistic backgrounds differ from that of the normative sample (68, 73). In addition, they may also fail to detect more subtle cognitive deficits that can cause distress in many patients (73).

**Table 5:** Comparison of two common cognitive impairment scales.

	**MoCA**	**FACT-COG**
General Description of measure	Assesses 9 cognitive domains: Attention, concentration, executive functions, memory, language, visuoconstructional skills, conceptual thinking, calculations, and orientation (68, 69)	37-item questionnaire made specifically for cancer survivors. Evaluates six cognitive domains: memory, concentration, mental acuity, verbal fluency, functional interference, and multitasking ability (70). Not to be associated with neuropsychological performance but rather depression and anxiety (71, 72).
Reported psychometric properties	Cronbach alpha: 0.8 Test-retest reliability was excellent, 0.91 (P < .001) with Good Internal consistency (73, 74).	Cronbach alpha was 0.86 (75, 76). The test-retest reliability was satisfactory with Intraclass correlation coefficient [ICC] of 0.762 (72).
Most common burden for clinical practice	Takes 10–15 min to complete (74).	Takes about 10–15 min to complete (77).
General scoring guidelines	One page 30-point test. - (3pt): Language - (5 pt) Visuospatial/ Executive functions. - (6pts) Attention, concentration, and working memory. - (3pt) Naming. - (2pts) Abstraction. - (5pts) short term memory. - (6pts) orientation to time and place (68, 73)	Out of 148 points with higher scores indicating better cognitive functioning. Perceived cognitive impairment (PCI) is defined as scores <54 using the 18 item version or score of <60 in 20 item version (78).
Clinical relevance	Good screening tool for all types of malignancy due to its ability to detect more subtle cognitive impairment (73, 74). While the brevity of the MoCA decreases the influence of patient fatigue on test results (74, 79, 80).	Unique tool to assess both cognitive concerns (impairment or deficiency) and cognitive abilities. Hence giving providers more information about cancer patient's cognitive complaint (75, 76).

*MoCA is the Montreal cognitive assessment scale; and FACT-COG is the functional assessment of cancer therapy-cognitive function*.

### Objective Measures

Strength, balance, mobility, and endurance are some of the important measures that rehabilitation providers look to assess carefully in their respective patient populations. Cancer rehabilitation specialists commonly need close assessments of these data points to better characterize functional capabilities, risk stratification, mortality prognostication, and QOL. Documentation of these data can vary greatly if done so on a subjective basis. However, special tests and instruments are described in Table 6, such as timed up and go (TUG) test, 5 times sit-to-stand (5XSST), and single-leg stance time (SLS) to create objective data points for providers to quantify and compare this data. In Table 6, we closely analyze the properties of common objective measures used in the cancer rehabilitation population and aim to individually assess the merit of each measure for continued use.

**Table 6:** Comparison of five of the most common cancer objective outcome measures.

	**Dynamometry**	**6MWT**	**TUG**	**5XSST**	**SLS**
General description of measure	An instrument that is used to measure hand grip strength (81).	Submaximal exercise test used to assess aerobic capacity and endurance (82).	Test used to assess a person's mobility and requires both static and dynamic balance (83).	A method to quantify lower extremity strength and/or identify movement strategies (84).	A test that can be effective in identifying individuals at risk of falling.
Reported psychometric properties	Cronbach Alpha: 0.95–0.98 (85).	Intraclass correlation coefficient *r* = 0.93 (86).	ICC = 0.97 (87).	ICC: 0.914–0.933 (88).	SLS performance with eyes open identified those with recent fall with a sensitivity of 0.83 (89).
Most common burden for clinical practice	Attention to detail required to ensure accuracy, a provider should be present for proper use.	Simply administered by a provider timing the subject.	Simply administered by a provider timing the subject.	Simply administered by a provider timing the subject.	Simply administered. Increased age and body mass index had a negative effect in following instructions (90).
General scoring guidelines	Scored using force production in kilograms or pounds. Weakness (grip strength <26 kg for men and <16 kg for women) (91).	Score is the distance in meters covered by the subject in 6 min.	Seconds it takes for a person to rise from a chair, walk three meters, walk back to the chair, and sit down (83).	Seconds it takes a subject to transfer from a seated to standing position and back to sitting five times (92).	Seconds a subject is able to stand on one leg with both eyes open and eyes closed up to 30 s (93).
Clinical relevance	Up for debate. Database of 500,000 showed weak hand grip correlates to poor health outcomes including some cancers (94).	To understand exercise capacity in rehabilitation populations including cancer populations (95).	Isolates tasks required for independent mobility and can be a predictor of complications in some cancer patients (96).	> 15 s identifies a risk of fall (97). More evaluations are needed for this test in cancer populations.	Cancer survivors impaired in their performance with eyes open demonstrated a decrease in QOL (89).

*6MWT is the 6-minute walk test; TUG is the timed up and go; 5XSST is the 5 times sit-to-stand; and SLS is the single-leg-stance time*.

## Conclusion

Outcome measures are a critical tool in assessing cancer patients before, during, and after cancer treatments. These assessments can include general function, QOL, pain, cognition, fatigue, and objective measures. These assessments not only monitor research outcomes but also assess a patient's positive and negative responses to interventions and safety to continue with cancer treatment. The outcome measures presented in this review are a small sampling of the available measures in the cancer rehabilitation setting. The author is optimistic that this review will provide the reader with a starting point in considering the useful outcome measures when starting a research project or focused patient assessment.

## Author Contributions

All authors contributed equal parts of the literature review, writing, and reviewing of this manuscript. This was a collaborative effort and teamwork under the guidance of EW.

## Conflict of Interest

The authors declare that the research was conducted in the absence of any commercial or financial relationships that could be construed as a potential conflict of interest.

## Publisher's Note

All claims expressed in this article are solely those of the authors and do not necessarily represent those of their affiliated organizations, or those of the publisher, the editors and the reviewers. Any product that may be evaluated in this article, or claim that may be made by its manufacturer, is not guaranteed or endorsed by the publisher.
